# Supplementary material for: Is social connectedness a risk factor for the spreading of COVID-19 among older adults? The Italian paradox
Source: PLoS One. 2020 May 21;15(5):e0233329. doi: 10.1371/journal.pone.0233329 (PMC7241742; doi:10.1371/journal.pone.0233329)
Supplement: Attachment — Submitted filename: rebuttal letter to the reviewers.docx [file pone.0233329.s001.docx]

Rome, 20/04/2020

To Prof P Abete

Academic Editor

Plos One

Dear Editor

Thank you for reviewing our paper “*Is social connectedness a risk factor for the spreading of COVID-19 among older adults? The Italian paradox”.* Please find below our answers to the reviewers. *In order to follow the PLOS ONE reproducibility criteria, demographic information and statistical method explanation have been added to the methodology sections. All the points raised by the reviewers have been addressed: a short paragraph have been added to the discussion according to the indication of the reviewer N-1. With regard to the concern raised by the reviewer N.2, unfortunately the requested information are not available to the public so far. We hope to have met all your requests in order to allow the publication of the article.*

*With my best regards*

*Prof. Giuseppe Liotta*

Reviewer 1

thank you for your answer. We now mention in the discussion the regional law you indicated with a short comment

Reviewer 2

Thank for your answer. There are data about COVID-19 related mortality and letality, however they are not yet provided as age-specific rate at regional level. This prevented us from any further analyses, since the paper is based on data fully available to the public. However, there are growing evidences of the dramatic impact on nursing home residents survival (see <https://www.epicentro.iss.it/coronavirus/pdf/sars-cov-2-survey-rsa-rapporto-2.pdf> also cited in the paper).
